# Optical Coherence Tomography: Retinal Imaging Contributes to the Understanding of Brain Pathology in Classical Galactosemia

**DOI:** 10.3390/jcm12052030

**Published:** 2023-03-03

**Authors:** Amelie S. Lotz-Havla, Tara Christmann, Klaus G. Parhofer, Esther M. Maier, Joachim Havla

**Affiliations:** 1 Department of Inborn Errors of Metabolism, Dr. von Hauner Children’s Hospital—University Hospital, LMU Munich, 80337 Munich, Germany; 2 Institute of Clinical Neuroimmunology, University Hospital, LMU Munich, 81377 Munich, Germany; 3 Medical Department IV-Grosshadern, University Hospital, LMU Munich, 81377 Munich, Germany; 4 Data Integration for Future Medicine (DIFUTURE) Consortium, LMU Munich, 81377 Munich, Germany

**Keywords:** classical galactosemia, optical coherence tomography (OCT), neurodegeneration, brain damage, retina, visual acuity

## Abstract

It remains unresolved whether central nervous system involvement in treated classical galactosemia (CG) is a progressive neurodegenerative process. This study aimed to investigate retinal neuroaxonal degeneration in CG as a surrogate of brain pathology. Global peripapillary retinal nerve fibre layer (GpRNFL) and combined ganglion cell and inner plexiform layer (GCIPL) were analysed in 11 CG patients and 60 controls (HC) using spectral–domain optical coherence tomography. Visual acuity (VA) and low-contrast VA (LCVA) were acquired to test visual function. GpRNFL and GCIPL did not differ between CG and HC (*p* > 0.05). However, in CG, there was an effect of intellectual outcome on GCIPL (*p* = 0.036), and GpRNFL and GCIPL correlated with neurological rating scale scores (*p* < 0.05). A single-case follow-up analysis showed GpRNFL (0.53–0.83%) and GCIPL (0.52–0.85%) annual decrease beyond the normal aging effect. VA and LCVA were reduced in CG with intellectual disability (*p* = 0.009/0.006), likely due to impaired visual perception. These findings support that CG is not a neurodegenerative disease, but that brain damage is more likely to occur early in brain development. To clarify a minor neurodegenerative component in the brain pathology of CG, we propose multicenter cross-sectional and longitudinal studies using retinal imaging.

## 1. Introduction

Classical galactosemia (CG, OMIM #230400) is an autosomal recessive disorder of galactose metabolism caused by bi-allelic variations in the *GALT* gene, leading to a galactose-1-phosphate uridyltransferase (GALT) enzyme activity of less than 1% [1]. Untreated patients with CG manifest in the newborn period with poor feeding, failure to thrive, jaundice, liver disease, cataracts, E. coli sepsis, and neonatal death [2]. Newborn screening for galactosemia enabling an early initiation of a galactose-restricted diet, prevents the life-threatening acute complications of the disease [3].

However, irrespective of timing of treatment initiation and degree of galactose restriction, most affected patients have an involvement of the central nervous system (CNS) [4,5,6,7]. The majority of the patients present with cognitive difficulties [8,9], speech and language disorders, including apraxia of speech or verbal dyspraxia [10,11,12], delay in social development [13,14], and motor dysfunction, such as abnormal muscular tone, tremors, and ataxia [15,16,17,18]. The long-term intellectual and neurological outcomes vary widely from normal to severely impaired, even within families with identical *GALT* gene variations [5].

The pathogenesis of CNS involvement in CG still remains unclear. There is an ongoing debate as to whether CG is a neurodegenerative disease caused by long-term exposure to endogenously produced galactose, poor dietary control, a combination of both, and/or other undetermined factors [19,20,21,22]. This is in contrast to the hypothesis that CNS involvement in CG is due to developmental abnormalities that are initiated in utero [15,23].

To understand whether CNS involvement in CG is a progressive neurodegenerative process or results from brain damage occurring early in brain development is crucial when considering new treatment strategies. To date, all potential new treatment strategies [24,25,26,27,28,29,30,31] aim to influence the postnatal effects of the disease. Subsequently, long-term sequelae that may be due to prenatal brain damage are not addressed.

Taken together, there is a need to better understand the pathogenesis of CNS involvement in early treated CG patients. This requires further creative research approaches to help clarify whether CG is a neurodegenerative disease.

One approach to study neurodegenerative processes in the CNS is to examine the retina [32]. Anatomically and developmentally, the retina is known as an extension of the CNS [32]. Several well defined neurodegenerative conditions that affect the brain also manifest in the retina [32]. The retina, therefore, has been postulated as a surrogate of brain health and a valuable model to study the CNS [33,34,35].

Optical coherence tomography (OCT) is a noninvasive examination technique of the retina that allows to visualize the different layers of the retina at very high resolution, thus providing a direct measure of neuronal integrity in neurodegenerative conditions [33,36,37,38]. The assessment of the peripapillary retinal nerve fiber layer (pRNFL), which contains unmyelinated axons, and the combined ganglion cell and inner plexiform layer (GCIPL), which contains retinal ganglion cell bodies, have been suggested as markers of subtle brain changes and have been used to monitor disease progression and aid in diagnostics of neurodegenerative diseases [39,40].

The aim of this study was to investigate by OCT whether patients with CG show signs of retinal neuroaxonal degeneration in comparison to a healthy control cohort. Furthermore, to investigate a possible association between axonal layers (pRNFL thickness) and neuronal layers (GCIPL volume) with intellectual and neurological outcome parameters.

## 2. Methods

### 2.1. Study Population

All patients who had been diagnosed with CG by neonatal or selective screening, and who were under regular care at the metabolic center of the Ludwig-Maximilians-University hospital in Munich, Germany, were invited to participate in this study. Inclusion criteria were confirmed CG and age six years and older. Exclusion criteria were: (i) ocular comorbidities potentially confounding the interpretation of OCT results (spherical equivalent >+5.5 diopters and <−5.5 diopters, astigmatism >+3 and <−3 diopters, history of ocular disease e.g. cataract, macular degeneration, glaucoma, and intracranial hypertension), (ii) history of systemic disease known to affect the retina, e.g. diabetes, (iii) history of any neurological disease unrelated to CG, (iv) prematurity <36 weeks of gestational age, and (v) current pregnancy. Exclusion criteria were identified based on the patients’ medical history.

An amount of 25 eligible patients were identified and offered to participate in the study. Thirteen patients decided to participate, and 11 of them met the inclusion criteria and were included in the study between October 2021 and June 2022 (see Table 1). Two patients could not be included due to exclusion criteria (cataract, recurrent headache). Sixty age matched healthy controls (HC) from our OCT database (NeuroVisionLab, LMU Hospital) were also included in the study.

The study was performed in accordance with the Helsinki II Declaration and approved by the ethics committee of the Ludwig-Maximilians-University of Munich, Medical Faculty (project no 19-0453). All participants and/or their legal representatives gave written informed consent.

### 2.2. Clinical Data

The following clinical data were obtained from the patients’ records (Table 1): method of diagnosis, age at start of treatment, genotype, GALT enzyme activity, erythrocyte galactose-1-phosphate (G1P) concentration at diagnosis, and lifetime G1P concentration. For the latter, the mean of all documented erythrocyte G1P concentrations after the age of 12 months was calculated. Measurements before 12 months of age were excluded, because, as expected [6], G1P concentrations in our cohort were relatively stable from 12 months of age, but not within the first year of life.

### 2.3. Outcome Data

To assess the intellectual outcome, information was obtained from the patient’s record. The individual’s school and professional career, everyday life skills, and the results of standardized age-specific intelligence tests if available were considered. The intelligence tests used were the Wechsler Preschool and Primary Scale of Intelligence, the Wechsler Intelligence Scale for Children, the Snijders-Oomen Non-verbal Intelligence test, the Kaufman Assessment Battery for Children, and the Wechsler Adult Intelligence Scale [41,42,43,44,45]. Patients were classified as intellectually disabled if they had an IQ < 75 [46], attended a special needs school, were employed in sheltered workshops, and were unable to live independently because of their cognitive impairment.

The neurological outcome was defined mainly by the presence or absence of movement disorder. A neurological examination, including a standardized tremor rating scale, the Fahn-Tolosa-Marin Clinical Rating Scale for tremor [47], and a standardized dystonia rating scale, the Fahn-Marsden Rating scale for dystonia [48], were performed by one trained examiner. In addition, data on speech development, ataxia, seizures, and the development of gross and fine motor skills reported by the treating physician were retrieved from the medical records.

### 2.4. Spectral-Domain Optical Coherence Tomography (SD-OCT)

OCT examination was performed using a SD-OCT (Spectralis, Heidelberg Engineering, Heidelberg, Germany) with automatic real time (ART) function for image averaging as described before [49]. The global pRNFL (GpRNFL) was measured with activated eye tracker using 3.4 mm ring scans around the optic nerve (12°, 1536 A-scans). Macular layers were calculated for a 6 mm diameter cylinder around the fovea from a macular volume scan (20° × 20°, 25 vertical B-scans). Segmentation of the ganglion cell (GCL) and inner plexiform layer (IPL) was performed semi-automatically using software provided by the OCT manufacturer (Eye Explorer 1.9.10.0 with viewing module 6.3.4.0, Heidelberg Engineering, Heidelberg, Germany). Based on the proposed consensus nomenclature [50,51], the combined GCIPL (GCL + IPL) volume was calculated (Supplementary Figure S1). The GCIPL thickness was derived from the GCIPL volume ×1000/9 × π. All scans were checked for sufficient quality and segmentation errors and corrected by the authors (TC), if necessary. OCT scans were acquired for both eyes in one participant. OCT data are reported according to the APOSTEL (2.0) and OSCAR-Ib recommendations [50,51,52].

**Table 1:** Demographic and disease-related information of classical galactosemia patients.

Patient No.	Gender	Age at Study [Years]	Method of Diagnosis	Start of Treatment [Days]	Genotype	GALT Enzyme Activity [µmol/h/g Hb]	G1P [mg/dL] at Diagnosis	Lifetime G1P [mg/dL]	Speech Disorder	Ataxia	Seizures	Intellectual Disability	Tremor ^#^ [Scaled Score]	Dystonia ^$^ [Scaled Score]
1	m	17	selective screening (newborn screening failure)	23	*p.Gln188Arg/* *p.Lys285Asn*	0	10.1	2.3	+	−	−	−	7	0
2	f	19	newborn screening	10	n.a.	0	43.0	2.4	+	+	−	+	53	9
3	f	21	family screening ^†^	0	*p.Gln188Arg/* *p.Gln188Arg*	0	5.0	2.1	+	−	−	+	7	0
4	m	23	newborn screening	8	*p.Gln188Arg/* *p.Gln188Arg*	0	33.5	2.4	−	−	−	−	15	0
5	f	25	family screening ^‡^	0	*p.Gln188Arg/* *p.Gln188Arg*	0	25.9	2.8	−	−	−	−	7	0
6	f	25	family screening ^†^	0	*p.Gln188Arg/* *p.Gln188Arg*	0	8.2	2.4	+	−	−	+	21	11
7	m	27	selective screening ^‡^	8	*p.Gln188Arg/* *p.Gln188Arg*	0	41.0	3.1	+	−	−	+	70	24.5
8	f	29	newborn screening ^†^	9	*p.Gln188Arg/* *p.Gln188Arg*	0	80.0	2.3	+	−	−	−	0	0
9	m	30	selective screening	9	*p.Gln188Arg/* unknown	0	67.7	2.1	−	−	−	−	10	11
10	f	36	newborn screening	11	n.a.	0	30.0	3.0	+	+	−	+	20	11.5
11	m	48	selective screening	14	*p.Gln188Arg/* *p.Gln212Ter*	n.a.	n.a.	1.9	+	+	+	+	54	6.5

Abbreviations: GALT; erythrocyte galactose-1-phosphate uridylyltranserase, G1P; galactose-1-phosphate, ^#^ Fahn-Tolosa-Marin Clinical Rating Scale for tremor (maximum possible total score of 144), ^$^ Fahn-Marsden Rating scale for dystonia (maximum possible total score of 120), n.a., not available, ^†^ first family, ^‡^ second family.

### 2.5. Visual Function Tests

Visual function tests were performed at the time of OCT examination. Visual acuity (VA) was tested with the standard Snellen chart. Habitually corrected low-contrast monocular visual acuity (LCVA) was acquired using 2.5% low-contrast Sloan letter charts placed in a retro-illuminated light box at 2 m distance. Each chart consisted of 14 lines with five letters per line that were standardized with equal difficulty per line and equal spacing between the lines. The total number of correct letters identified on each chart was tested to determine LCVA (maximum 70 letters). VA and LCVA were tested for both eyes in one participant.

### 2.6. Statistical Analyses

Statistical analyses were performed using SPSS Statistics 26 (IBM) by the lead author (ASL-H).

Comparison of demographic data between the patient and control group was analyzed by chi-square test. To analyze an association between clinical data and clinical outcome parameters of CG patients, bivariate correlation analysis was performed, and subgroups were compared using the Kruskal-Wallis test.

Mean values (mean) and standard errors (SE) of SD-OCT data and VA/LCVA were calculated using a linear mixed effects model, which accounted for clustering because two eyes were evaluated for each participant. The effects of disease status, age, and intellectual disability on SD-OCT data and VA/LCVA were evaluated using generalized estimating equation (GEE) regression models to account for within-patient inter-eye correlation. The correlation matrix parameter was set to ‘exchangeable’. In these models, SD-OCT data and VA/LCVA were the dependent variables, while patient’s disease status and/or intellectual outcome status were the independent variable. Age was added as independent covariate. To evaluate the association of SD-OCT data and neurological rating scale scores within the CG cohort, partial correlation analysis controlling for age was applied.

For all analyses, *p*-values ≤ 0.05 were considered significant.

## 3. Results

### 3.1. Patients Characteristics and Disease Features

Eleven CG patients (age 17–48 years, mean ± SD; 27.3 ± 8.5 years) were included in the study. The CG cohort included two families with two and three siblings, respectively. The matching of patients and controls based on the age resulted in 60 HC (age 16–50 years, mean ± SD 28.9 ± 8.9 years, χ^2^ = 46.3, *p* = 0.338).

Demographic data and disease-related parameters of the CG patients are shown in Table 1. Four patients were diagnosed by selective screening within the newborn period after the onset of disease-typical symptoms. Four patients were diagnosed by newborn screening. At the time of admission to the metabolic center, all four patients showed signs and symptoms of GC ranging from jaundice to liver failure, tubulopathy, and incipient cataract, all completely resolved during the course of treatment. The remaining three patients were diagnosed by family screening before the onset of symptoms. In all patients, treatment was started within the newborn period between birth and 23 days.

The diagnosis of CG was confirmed by genotype and/or enzymatic analysis. Genotyping was available in nine of the eleven patients and showed the prevalent pathogenic variant p.Gln188Arg as either homozygous (N = 6) or in a compound-heterozygous state with the pathogenic variants p.Lys285Asn (N = 1) or p.Gln212Ter (N = 1). GALT enzyme activity in erythrocytes was available in 10 of the eleven patients and was below the limit of quantification in all of them.

All patients had elevated G1P concentrations at diagnosis (range 5–80 mg/dL). Of note, three patients received lactose-free formula from birth due to a positive family history. All patients followed dietary treatment consequently throughout life, demonstrated by lifetime G1P concentrations within the therapeutic range (range 1.9–3.1 mg/d, mean ± SD 2.4 ± 0.4 mg/dL) [53,54].

Concerning the long-term clinical outcome, six patients were categorized as intellectually disabled. Of the remaining five patients, three had been found to have a low-average IQ (80–89), three needed additional support during their school career, and all pursued occupations that did not require higher cognitive performance.

A total of ten patients were diagnosed with movement disorders ranging from tremor only (N = 4) to tremor with additional dystonia (N = 6) and ataxia (N = 3) affecting activities of daily living. One patient was treated for seizures. Speech disorders ranging from developmental speech delay to scanning speech and verbal dyspraxia were reported in eight patients.

As expected, there were no significant correlations between time of treatment start or G1P concentration at diagnosis and tremor (start of treatment; r = 0.144, *p* = 0.672/G1P concentration at diagnosis; r = −0.034, *p* = 0.931) or dystonia (start of treatment; r = 0.094, *p* = 0.782/G1P concentration at diagnosis; r = −0.220, *p* = 0.569) rating scale scores or the intellectual outcome (start of treatment; r = −0.048, *p* = 0.888/G1P concentration at diagnosis; r = −0.178, *p* = 0.646). Tremor (*p* = 0.578) and dystonia (*p* = 0.624) rating scale scores were not significantly different in CG patients subgrouped according to the method of diagnosis (family screening, newborn screening, or selective screening).

### 3.2. SD-OCT Parameters Related to Disease Status and Age

Twenty-two eyes of eleven CG patients were compared with 119 eyes of 60 HC.

There was no significant effect of disease status on the GpRNFL thickness and the GCIPL volume (for both *p* > 0.05) (Table 2). Furthermore, there was no significant interaction effect of disease status and age on GpRNLF thickness and on GCIPL volume (for both *p* > 0.05) (Table 2). The range of GpRNFL thickness of all CG patients (86–112 µm) was within the range of the HC cohort (85–128 µm). The volumes of GCIPL were below the HC range in N = 2 eyes of one patient (CG vs. HC range 1.59–2.28 mm^3^ vs. 1.76–2.40 mm^3^ corresponding to a GCIPL thickness of 56.2–80.6 µm vs. 62.3–84.9 µm).

### 3.3. SD-OCT Parameters Related to Clinical Outcome

**Figure 1:**
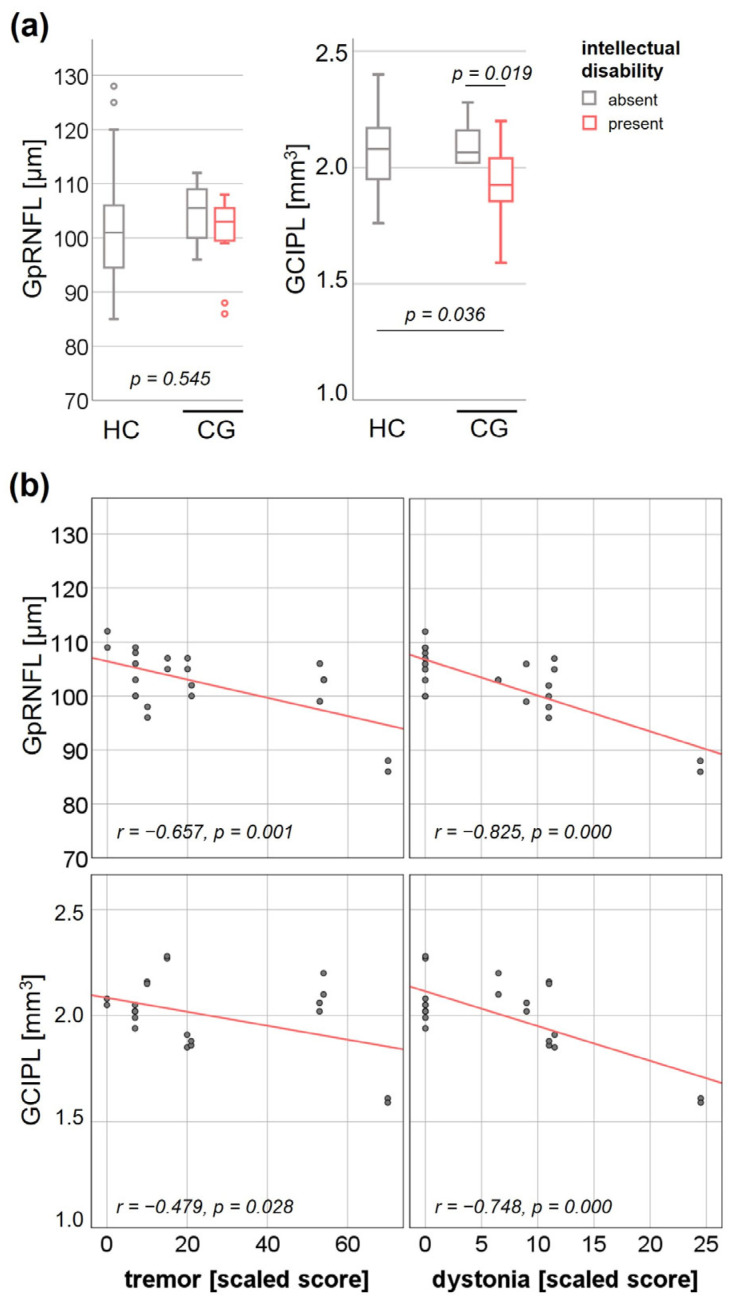
Effect of clinical outcome on global peripapillary retinal nerve fibre layer (GpRNFL) thickness and combined ganglion cell and inner plexiform layer (GCIPL) volume. (**a**) Interaction effect of diagnosis (HC; healthy controls, CG; classical galactosemia) and intellectual outcome on GpRNFL thickness and GCIPL volume. (**b**) Association of GpRNFL thickness and GCIPL volume with the Fahn-Tolosa-Marin Clinical Rating Scale scores for tremor and the Fahn-Marsden Rating Scale scores for dystonia.

GEE analysis with disease status and intellectual outcome as independent variables showed no interaction effect on GpRNFL thickness (Wald Chi-square 2.13, *p* = 0.545) (Figure 1a). As the number of CG patients without any movement disorder was limited to two eyes from one patient resulting in inadequate power, cross-sectional subgroup analysis with neurological outcome as independent variable was not performed. Partial correlation analysis adjusted for age resulted in significant negative correlations between GpRNFL thickness and tremor (r = −0.657, *p* = 0.001) and dystonia (r = −0.825, *p* = 0.000) rating scale scores (Figure 1b).

Regarding GCIPL volumes, there was a significantly reduced GCIPL volume in those CG patients with intellectual disability (mean ± SE 1.92 ± 0.07, 95% CI 1.8–2.05) compared to HC (mean ± SE 2.07 ± 0.02, 95% CI 2.03–2.1, *p* = 0.036) and CG without intellectual disability (mean ± SE 2.11 ± 0.04, 95% CI 1.8–2.05, *p* = 0.019) (Figure 1a). Partial correlation analysis adjusted for age resulted in a significant negative association between the GCIPL volume and tremor (r = −0.479, *p* = 0.028) and dystonia (r = −0.748, *p* = 0.000) rating scale scores (Figure 1b).

### 3.4. Longitudinal Single Case Observation

In one single-case (patient No. 2), we had the opportunity to perform SD-OCT follow-up analysis after 5.7 years. A comprehensive neurological workup performed as part of clinical care was available in the records of this patient. Cranial magnetic resonance imaging revealed frontal and parietal white matter hyperintensities, subcortical abnormal cerebral white matter signal intensity, as well as cerebellar atrophy, all findings consistent with CG. Cerebrospinal fluid examination revealed no evidence of an inflammatory abnormality or barrier disruption.

Comparing baseline SD-OCT data and follow-up, GpRNFL decreased by right 0.88/left 0.52 µm per year (annual decrease of right 0.83%/left 0.53%) between 19 and 25 years of age (Table 3, Supplementary Figure S2). The GCIPL volume decreased by right 0.01/left 0.02 mm^3^, corresponding to an annual decrease in thickness of right 0.36/left 0.63 µm (annual decrease of right 0.52%/left 0.85%) (Table 3, Supplementary Figure S2).

### 3.5. VA/LCVA Related to Disease Status and Intellectual Outcome

Twenty eyes of eleven CG patients were evaluated compared to 56 eyes of HC. One CG patient (two eyes) had to be excluded because he could not read and was unable to participate in the examination due to cognitive impairment. Visual acuity was not available in all HC included in the study.

In the CG cohort, VA (mean ± SE 0.66 ± 0.08) and LCVA (mean ± SE 25.6 ± 3.24) were significantly lower compared to the HC cohort (VA; mean ± SE 0.87 ± 0.03, *p* = 0.009, LCVA; mean ± SE 34.8 ± 1.2, *p* = 0.006). GEE analysis revealed a significant interaction effect of disease status and intellectual status on VA and LCVA (Table 4), with a significantly lower VA and LCVA in those CG patients with intellectual disability.

## 4. Discussion

To contribute to the unresolved issue of whether CNS involvement in treated CG is a progressive neurodegenerative process or a consequence of brain damage occurring early in brain development [15,23], we examined CG patients for signs of neuroaxonal degeneration using retinal imaging by SD-OCT. Overall, our data show no evidence of retinal neuroaxonal degeneration in treated GC patients as a surrogate of neurodegenerative disease compared to HC. However, we found significant associations between GpRNFL thickness and GCIPL volume in CG patients with intellectual and/or neurological outcome parameters.

Based on current knowledge, we hypothesize that, if GC was a neurodegenerative disease, we would observe significant atrophy of GpRNFL and GCIPL compared with age-matched HC, depending on disease status and duration. [39,40]. However, the GpRNFL thickness was within the range of our HC cohort for all CG patients included in the study and there was no significant effect of disease status on GpRNFL thickness. Supporting this, the mean global pRNFL thickness of our CG cohort was comparable to the mean pRNFL thickness of larger normative datasets in the literature [55,56]. There was also no significant difference in GCIPL volume between CG patients and our HC cohort, and the mean GCIPL volume (or corresponding mean GCIPL thickness) was comparable to that of large normative data sets [55,56]. Taken together, we could not show evidence of relevant retinal neuroaxonal degeneration for the group of patients with GC, neither in comparison to our healthy control cohort nor in comparison to published normative databases, although all of our patients had intellectual and/or neurological complications.

Thus, according to our hypothesis, our findings do not suggest that CG is a neurodegenerative disease. Rather, they support that brain damage occurs early during brain development [15]: the major morphologic development of the retina takes place prenatally and the newborn retina is composed of the different layers as in adults. However, the maturation of the retina occurs postnatally [57,58], and it has been demonstrated in vivo that central retinal and foveal thickness increase logarithmically between birth and four years of age, and foveal development is not complete until the age of 12 years [59]. Therefore, potential effects of brain damage that occur early in brain development might be minimized by the ability of the retina to develop until adolescence.

Alternative explanations for our findings, which show no evidence of relevant retinal neuroaxonal degeneration, could be that the neurodegeneration in GC is not generalized but is restricted to specific brain regions (e.g., cerebellum, basal ganglia) or that the CNS involvement in CG is due to functional disorders, both pathologies that are not detected by retinal imaging. However, we consider it unlikely that these are the sole explanations for our findings, as decreased cerebral grey and white matter volume has been found in CG [21].

Although we did not find evidence of retinal neuroaxonal degeneration in our CG cohort compared to the HC cohort, there was a significant correlation of GpRNFL thickness and neurological (tremor, dystonia) rating scale scores. The correlation effect was mainly driven by two eyes of one patient who had the most marked movement disorder. For the GCIPL, findings were more pronounced: the same patient had a GCIPL volume below the HC range and there was a significant association of GCIPL volumes in the overall CG cohort not only with the neurological rating scale scores for tremor and dystonia but also the intellectual outcome. For other neurodegenerative diseases, it has been demonstrated that the GCIPL is superior to distinguish affected and healthy individuals [60,61,62,63]. Furthermore, we had the opportunity to perform a SD-OCT follow-up study in one single CG patient with intellectual disability and marked movement disorder. This revealed an annual decrease in GpRNFL and GCIPL thickness that was higher than the proportion of GpRNFL and GCIPL thinning attributed to normal aging in published healthy cohorts [64,65]. Whether these observations point to a partial progressive neurodegenerative component in the pathogenesis of CNS involvement in CG is merely speculative based on this pilot research project. However, our data motivate multicentre cross-sectional and longitudinal studies in larger CG cohorts to further evaluate neurodegeneration in CG using the retinal model.

Results of visual function revealed a significantly reduced VA and LCVA in CG patients with intellectual disability. This outcome may be biased by impairments in visual perception and spatial processing, as has been demonstrated for patients with CG [66,67,68]. To our knowledge, visual function impairment independent of cataract has not been described in patients with CG, a patient population that is regularly monitored by ophthalmologists [3,69,70]. Considering this, the poor performance of CG patients with intellectual disability in terms of VA and LCVA observed in the present study is likely due to cognitive dysfunction per se rather than decreased visual dysfunction.

### Limitations of the Study

The main limitation of this study is the relatively small sample size due to the low prevalence of this rare disease, which is about 1:77,000 in Germany [71]. This may have resulted in an underestimation of a possible small effect (considering the distribution of the GCIPL volume in our CG cohort) or an overestimation of random distribution (considering the correlation between pRNFL thickness or GCIPL volume and neurological rating scale scores).

Another limitation is that standardized MRI scans were not included in this study. However, abnormal cerebral white matter signal intensity, white matter hyperintensities, cerebral atrophy, and cerebellar atrophy have repeatedly been described in patients with CG with the underlying molecular mechanisms remaining elusive [19,21,67,72,73,74].

## 5. Conclusions

This exploratory study of retinal imaging in CG patients supports the hypothesis that CNS involvement in treated CG patients is not primarily a progressive neurodegenerative process, but that brain damage is more likely to occur early in brain development. If this holds true, it will raise questions about future treatment approaches in CG with respect to CNS involvement, as all new treatment strategies to date aim to influence the postnatal effects of the disease. However, given the significance of our main statement, it is important to emphasize that we cannot exclude a minor neurodegenerative component in the pathogenesis of CNS involvement in CG. Multicenter cross-sectional studies on larger CG cohorts and longitudinal research that reveals the course of retinal neuroaxonal degeneration parameters could clarify a partial contribution of progressive neurodegeneration to the pathogenesis of CNS involvement in CG. For this purpose, we propose retinal imaging by SD-OCT as a sensitive, reliable, safe, low-burden, low-cost, and easy-to-use tool.

## Figures and Tables

**Table 2:** Optical coherence tomography findings of classical galactosemia patients compared to healthy controls.

SD-OCT Parameters	HC		CG		Effect Disease Status		Effect Disease Status × Age	
	N = 119		N = 22					
	Mean ± SE	95% CI	Mean ± SE	95% CI	Wald Chi-Square	*p*	Wald Chi-Square	*p*
GpRNFL [µm]	100.8 ± 1.0	98.8–102.8	102.4 ± 1.8	98.8–105.9	0.62	0.431	0.31	0.579
GCIPL [mm^3^]	2.07 ± 0.02	2.03–2.1	2.01 ± 0.05	1.9–2.1	0.70	0.401	2.82	0.244

Abbreviations: SD-OCT; spectral-domain optical coherence tomography, HC; healthy controls, CG; classical galactosemia patients, N; number of eyes, GpRNFL; global peripapillary retinal nerve fiber layer, GCIPL; ganglion cell and inner plexiform layer. SE; standard error, 95% CI; 95% confidence interval.

**Table 3:** Longitudinal single-case observation of change in optical coherence tomography parameters.

SD-OCT Parameters		Baseline	Follow-up	Annual Decrease	Annual % Decrease
GpRNFL [µm]	right eye	106	101	−0.88	−0.83
	left eye	99	96	−0.53	−0.53
GCIPL [mm^3^/(µm)]	right eye	2.02 (71.4)	1.96 (69.3)	−0.01 (−0.37)	−0.52
	left eye	2.06 (72.9)	1.96 (69.3)	−0.02 (−0.63)	−0.85

Abbreviations: SD-OCT; spectral-domain optical coherence tomography, GpRNFL; global peripapillary retinal nerve fiber layer, GCIPL; ganglion cell and inner plexiform layer.

**Table 4:** Visual acuity of classical galactosemia patients compared to healthy controls.

	HC	CG				Effect Disease Status × Intellectual Disability	
		Without Intellectual Disability		With Intellectual Disability			
	Mean ± SE	Mean ± SE	*p*	Mean ± SE	*p*	Wald Chi-Square	*p*
VA	0.87 ± 0.03	0.84 ± 0.05	0.613	0.48 ± 0.26	<0.001 *	13.5	0.001 *
LCVA [raw letters]	34.8 ± 1.2	34.2 ± 1.9	0.787	17.0 ± 7.63	<0.001 *	30.8	<0.001 *

Abbreviations: HC; healthy controls, CG; classical galactosemia patients, VA; visual acuity, LCVA; low-contrast visual acuity, SE; standard error, * *p* < 0.05.

## Data Availability

All data generated or analyzed during this study are included in this published article.

## Supplementary Materials

The following supporting information can be downloaded at: https://www.mdpi.com/article/10.3390/jcm12052030/s1, Figure S1: Spectral-domain optical coherence tomography macular scan with retinal structures highlighted according to the consensus nomenclature.; Figure S2: Baseline and follow-up spectral-domain optical coherence tomography in patient No. 2.

## References

[1] Succoio M., Sacchettini R., Rossi A., Parenti G., Ruoppolo M. (2022). Galactosemia: Biochemistry, Molecular Genetics, Newborn Screening, and Treatment. Biomolecules.

[2] Demirbas D., Coelho A.I., Rubio-Gozalbo M.E., Berry G.T. (2018). Hereditary galactosemia. Metabolism.

[3] Rubio-Gozalbo M.E., Haskovic M., Bosch A.M., Burnyte B., Coelho A.I., Cassiman D., Couce M.L., Dawson C., Demirbas D., Derks T. (2019). The natural history of classic galactosemia: Lessons from the GalNet registry. Orphanet J. Rare Dis..

[4] Delnoy B., Coelho A.I., Rubio-Gozalbo M.E. (2021). Current and Future Treatments for Classic Galactosemia. J. Pers. Med..

[5] Hughes J., Ryan S., Lambert D., Geoghegan O., Clark A., Rogers Y., Hendroff U., Monavari A., Twomey E., Treacy E.P. (2009). Outcomes of siblings with classical galactosemia. J. Pediatr..

[6] Welsink-Karssies M.M., Ferdinandusse S., Geurtsen G.J., Hollak C.E.M., Huidekoper H.H., Janssen M.C.H., Langendonk J.G., van der Lee J.H., O’Flaherty R., Oostrom K.J. (2020). Deep phenotyping classical galactosemia: Clinical outcomes and biochemical markers. Brain Commun..

[7] Kuiper A., Grunewald S., Murphy E., Coenen M.A., Eggink H., Zutt R., Rubio-Gozalbo M.E., Bosch A.M., Williams M., Derks T.G.J. (2019). Movement disorders and nonmotor neuropsychological symptoms in children and adults with classical galactosemia. J. Inherit. Metab. Dis..

[8] Waggoner D.D., Buist N.R., Donnell G.N. (1990). Long-term prognosis in galactosaemia: Results of a survey of 350 cases. J. Inherit. Metab. Dis..

[9] Schadewaldt P., Hoffmann B., Hammen H.W., Kamp G., Schweitzer-Krantz S., Wendel U. (2010). Longitudinal assessment of intellectual achievement in patients with classical galactosemia. Pediatrics.

[10] Waisbren S.E., Norman T.R., Schnell R.R., Levy H.L. (1983). Speech and language deficits in early-treated children with galactosemia. J. Pediatr..

[11] Nelson C.D., Waggoner D.D., Donnell G.N., Tuerck J.M., Buist N.R. (1991). Verbal dyspraxia in treated galactosemia. Pediatrics.

[12] Potter N.L., Nievergelt Y., Shriberg L.D. (2013). Motor and speech disorders in classic galactosemia. JIMD Rep..

[13] Bosch A.M., Maurice-Stam H., Wijburg F.A., Grootenhuis M.A. (2009). Remarkable differences: The course of life of young adults with galactosaemia and PKU. J. Inherit. Metab. Dis..

[14] Gubbels C.S., Maurice-Stam H., Berry G.T., Bosch A.M., Waisbren S., Rubio-Gozalbo M.E., Grootenhuis M.A. (2011). Psychosocial developmental milestones in men with classic galactosemia. J. Inherit. Metab. Dis..

[15] Ahtam B., Waisbren S.E., Anastasoaie V., Berry G.T., Brown M., Petrides S., Afacan O., Prabhu S.P., Schomer D., Grant P.E. (2020). Identification of neuronal structures and pathways corresponding to clinical functioning in galactosemia. J. Inherit. Metab. Dis..

[16] Waisbren S.E., Potter N.L., Gordon C.M., Green R.C., Greenstein P., Gubbels C.S., Rubio-Gozalbo E., Schomer D., Welt C., Anastasoaie V. (2012). The adult galactosemic phenotype. J. Inherit. Metab. Dis..

[17] Rubio-Agusti I., Carecchio M., Bhatia K.P., Kojovic M., Parees I., Chandrashekar H.S., Footitt E.J., Burke D., Edwards M.J., Lachmann R.H. (2013). Movement disorders in adult patients with classical galactosemia. Mov. Disord..

[18] Coelho A.I., Rubio-Gozalbo M.E., Vicente J.B., Rivera I. (2017). Sweet and sour: An update on classic galactosemia. J. Inherit. Metab. Dis..

[19] Rossi-Espagnet M.C., Sudhakar S., Fontana E., Longo D., Davison J., Petengill A.L., Bevivino E., Pacheco F.T., da Rocha A.J., Hanagandi P. (2021). Neuroradiologic Phenotyping of Galactosemia: From the Neonatal Form to the Chronic Stage. Am. J. Neuroradiol..

[20] Hermans M.E., van Weeghel M., Vaz F.M., Ferdinandusse S., Hollak C.E.M., Huidekoper H.H., Janssen M.C.H., van Kuilenburg A.B.P., Pras-Raves M.L., Wamelink M.M.C. (2022). Multi-omics in classical galactosemia: Evidence for the involvement of multiple metabolic pathways. J. Inherit. Metab. Dis..

[21] Welsink-Karssies M.M., Schrantee A., Caan M.W.A., Hollak C.E.M., Janssen M.C.H., Oussoren E., de Vries M.C., Roosendaal S.D., Engelen M., Bosch A.M. (2020). Gray and white matter are both affected in classical galactosemia: An explorative study on the association between neuroimaging and clinical outcome. Mol. Genet. Metab..

[22] Randall J.A., Sutter C., Wang S., Bailey E., Raither L., Perfetti R., Shendelman S., Burbridge C. (2022). Qualitative interviews with adults with Classic Galactosemia and their caregivers: Disease burden and challenges with daily living. Orphanet J. Rare Dis..

[23] Fridovich-Keil J.L., Berry G.T. (2022). Pathophysiology of long-term complications in classic galactosemia: What we do and do not know. Mol. Genet. Metab..

[24] Daenzer J.M.I., Rasmussen S.A., Patel S., McKenna J., Fridovich-Keil J.L. (2022). Neonatal GALT gene replacement offers metabolic and phenotypic correction through early adulthood in a rat model of classic galactosemia. J. Inherit. Metab. Dis..

[25] Timson D.J. (2020). Therapies for galactosemia: A patent landscape. Pharm. Pat. Anal..

[26] Banford S., McCorvie T.J., Pey A.L., Timson D.J. (2021). Galactosemia: Towards Pharmacological Chaperones. J. Pers. Med..

[27] Liguori L., Monticelli M., Allocca M., Hay Mele B., Lukas J., Cubellis M.V., Andreotti G. (2020). Pharmacological Chaperones: A Therapeutic Approach for Diseases Caused by Destabilizing Missense Mutations. Int. J. Mol. Sci..

[28] Balakrishnan B., An D., Nguyen V., DeAntonis C., Martini P.G.V., Lai K. (2020). Novel mRNA-Based Therapy Reduces Toxic Galactose Metabolites and Overcomes Galactose Sensitivity in a Mouse Model of Classic Galactosemia. Mol. Ther..

[29] Rasmussen S.A., Daenzer J.M.I., Fridovich-Keil J.L. (2021). A pilot study of neonatal GALT gene replacement using AAV9 dramatically lowers galactose metabolites in blood, liver, and brain and minimizes cataracts in GALT-null rat pups. J. Inherit. Metab. Dis..

[30] Delnoy B., Haskovic M., Vanoevelen J., Steinbusch L.K.M., Vos E.N., Knoops K., Zimmermann L.J.I., Noga M., Lefeber D.J., Martini P.G.V. (2022). Novel mRNA therapy restores GALT protein and enzyme activity in a zebrafish model of classic galactosemia. J. Inherit. Metab. Dis..

[31] Brophy M.L., Stansfield J.C., Ahn Y., Cheng S.H., Murphy J.E., Bell R.D. (2022). AAV-mediated expression of galactose-1-phosphate uridyltransferase corrects defects of galactose metabolism in classic galactosemia patient fibroblasts. J. Inherit. Metab. Dis..

[32] London A., Benhar I., Schwartz M. (2013). The retina as a window to the brain-from eye research to CNS disorders. Nat. Rev. Neurol..

[33] Moran C., Xu Z.Y., Mehta H., Gillies M., Karayiannis C., Beare R., Chen C., Srikanth V. (2022). Neuroimaging and cognitive correlates of retinal Optical Coherence Tomography (OCT) measures at late middle age in a twin sample. Sci. Rep..

[34] Cheung C.Y., Chan V.T.T., Mok V.C., Chen C., Wong T.Y. (2019). Potential retinal biomarkers for dementia: What is new?. Curr. Opin. Neurol..

[35] Chan V.T.T., Tso T.H.K., Tang F., Tham C., Mok V., Chen C., Wong T.Y., Cheung C.Y. (2017). Using Retinal Imaging to Study Dementia. JoVE (J. Vis. Exp.).

[36] Galetta K.M., Calabresi P.A., Frohman E.M., Balcer L.J. (2011). Optical coherence tomography (OCT): Imaging the visual pathway as a model for neurodegeneration. Neurotherapeutics.

[37] Albrecht P., Muller A.K., Sudmeyer M., Ferrea S., Ringelstein M., Cohn E., Aktas O., Dietlein T., Lappas A., Foerster A. (2012). Optical coherence tomography in parkinsonian syndromes. PLoS ONE.

[38] Cunha L.P., Almeida A.L., Costa-Cunha L.V., Costa C.F., Monteiro M.L. (2016). The role of optical coherence tomography in Alzheimer’s disease. Int. J. Retin. Vitr..

[39] Xie J., Donaldson L., Margolin E. (2022). The use of optical coherence tomography in neurology: A review. Brain.

[40] Airen S., Shi C., Liu Z., Levin B.E., Signorile J.F., Wang J., Jiang H. (2020). Focal alteration of the intraretinal layers in neurodegenerative disorders. Ann. Eye Sci..

[41] Tellegen P.J., Winkel M., Wijnberg-Williams B.J., Laros J.A. (1998). Snijders-Oomen Nonverbal Intelligence Test.

[42] Lichtenberger E.O., Sotelo-Dynega M., Kaufman A.S., Naglieri J.A., Goldstein S. (2009). The Kaufman Assessment Battery for Children—Second Edition. Practitioner’s Guide to Assessing Intelligence and Achievement.

[43] Wechsler D. (2008). Wechsler Adult Intelligence Scale—Fourth Edition (WAIS-IV).

[44] Wechsler D. (1989). Wechsler Preschool and Primary Scale of Intelligence—Revised.

[45] Wechsler D. (1949). Wechsler Intelligence Scale for Children. J. Consult. Psychol..

[46] American Psychiatric Association (APA) (2022). Diagnostic and Statistical Manual of Mental Disorders. Diagnostic Features DSM-5-TR.

[47] Fahn S., Tolosa E., Conceppcion M., Jankovic J., Tolosa E. (1993). Clinical rating scale for tremor. Parkinson’s Disease and Movement Disorders.

[48] Burke R.E., Fahn S., Marsden C.D., Bressman S.B., Moskowitz C., Friedman J. (1985). Validity and reliability of a rating scale for the primary torsion dystonias. Neurology.

[49] Lotz-Havla A.S., Weiss K., Schiergens K., Regenauer-Vandewiele S., Parhofer K.G., Christmann T., Bohm L., Havla J., Maier E.M. (2021). Optical Coherence Tomography to Assess Neurodegeneration in Phenylalanine Hydroxylase Deficiency. Front. Neurol..

[50] Aytulun A., Cruz-Herranz A., Aktas O., Balcer L.J., Balk L., Barboni P., Blanco A.A., Calabresi P.A., Costello F., Sanchez-Dalmau B. (2021). The APOSTEL 2.0 Recommendations for Reporting Quantitative Optical Coherence Tomography Studies. Neurology.

[51] Cruz-Herranz A., Balk L.J., Oberwahrenbrock T., Saidha S., Martinez-Lapiscina E.H., Lagreze W.A., Schuman J.S., Villoslada P., Calabresi P., Balcer L. (2016). The APOSTEL recommendations for reporting quantitative optical coherence tomography studies. Neurology.

[52] Schippling S., Balk L.J., Costello F., Albrecht P., Balcer L., Calabresi P.A., Frederiksen J.L., Frohman E., Green A.J., Klistorner A. (2015). Quality control for retinal OCT in multiple sclerosis: Validation of the OSCAR-IB criteria. Mult. Scler..

[53] Shin Y.S., Zschocke J., Das A.M., Podskarbi T. (1999). Molecular and biochemical basis for variants and deficiency forms of galactose-1-phosphate uridyltransferase. J. Inherit. Metab. Dis..

[54] Berry G.T., Adam M.P., Everman D.B., Mirzaa G.M., Pagon R.A., Wallace S.E., Bean L.J.H., Gripp K.W., Amemiya A. (1993). Classic Galactosemia and Clinical Variant Galactosemia. GeneReviews((R)).

[55] Meyer J., Karri R., Danesh-Meyer H., Drummond K., Symons A. (2021). A Normative Database of A-Scan Data Using the Heidelberg Spectralis 4 Spectral Domain Optical Coherence Tomography Machine. PLoS ONE.

[56] Mauschitz M.M., Holz F.G., Finger R.P., Breteler M.M.B. (2019). Determinants of Macular Layers and Optic Disc Characteristics on SD-OCT: The Rhineland Study. Transl. Vis. Sci. Technol..

[57] Smelser G.K., Ozanics V., Rayborn M., Sagun D. (1974). Retinal synaptogenesis in the primate. Investig. Ophthalmol. Vis. Sci..

[58] Van Cruchten S., Vrolyk V., Perron Lepage M.F., Baudon M., Voute H., Schoofs S., Haruna J., Benoit-Biancamano M.O., Ruot B., Allegaert K. (2017). Pre- and Postnatal Development of the Eye: A Species Comparison. Birth Defects Res..

[59] Lee H., Purohit R., Patel A., Papageorgiou E., Sheth V., Maconachie G., Pilat A., McLean R.J., Proudlock F.A., Gottlob I. (2015). In Vivo Foveal Development Using Optical Coherence Tomography. Investig. Ophthalmol. Vis. Sci..

[60] Almeida A.L.M., Pires L.A., Figueiredo E.A., Costa-Cunha L.V.F., Zacharias L.C., Preti R.C., Monteiro M.L.R., Cunha L.P. (2019). Correlation between cognitive impairment and retinal neural loss assessed by swept-source optical coherence tomography in patients with mild cognitive impairment. Alzheimer’s Dement. Diagn. Assess. Dis. Monit..

[61] Lopez-Cuenca I., de Hoz R., Salobrar-Garcia E., Elvira-Hurtado L., Rojas P., Fernandez-Albarral J.A., Barabash A., Salazar J.J., Ramirez A.I., Ramirez J.M. (2020). Macular Thickness Decrease in Asymptomatic Subjects at High Genetic Risk of Developing Alzheimer’s Disease: An OCT Study. J. Clin. Med..

[62] Salobrar-Garcia E., Hoyas I., Leal M., de Hoz R., Rojas B., Ramirez A.I., Salazar J.J., Yubero R., Gil P., Trivino A. (2015). Analysis of Retinal Peripapillary Segmentation in Early Alzheimer’s Disease Patients. BioMed Res. Int..

[63] Cheung C.Y., Ong Y.T., Hilal S., Ikram M.K., Low S., Ong Y.L., Venketasubramanian N., Yap P., Seow D., Chen C.L. (2015). Retinal ganglion cell analysis using high-definition optical coherence tomography in patients with mild cognitive impairment and Alzheimer’s disease. J. Alzheimer’s Dis..

[64] Kenney R., Liu M., Hasanaj L., Joseph B., Al-Hassan A.A., Balk L., Behbehani R., Brandt A.U., Calabresi P.A., Frohman E.M. (2022). Normative Data and Conversion Equation for Spectral-Domain Optical Coherence Tomography in an International Healthy Control Cohort. J. Neuroophthalmol..

[65] Sotirchos E.S., Gonzalez Caldito N., Filippatou A., Fitzgerald K.C., Murphy O.C., Lambe J., Nguyen J., Button J., Ogbuokiri E., Crainiceanu C.M. (2020). Progressive Multiple Sclerosis Is Associated with Faster and Specific Retinal Layer Atrophy. Ann. Neurol..

[66] Hermans M.E., Welsink-Karssies M.M., Bosch A.M., Oostrom K.J., Geurtsen G.J. (2019). Cognitive functioning in patients with classical galactosemia: A systematic review. Orphanet J. Rare Dis..

[67] Kaufman F.R., McBride-Chang C., Manis F.R., Wolff J.A., Nelson M.D. (1995). Cognitive functioning, neurologic status and brain imaging in classical galactosemia. Eur. J. Pediatr..

[68] Doyle C.M., Channon S., Orlowska D., Lee P.J. (2010). The neuropsychological profile of galactosaemia. J. Inherit. Metab. Dis..

[69] Burke J.P., O’Keefe M., Bowell R., Naughten E.R. (1989). Ophthalmic findings in classical galactosemia—A screened population. J. Pediatr. Ophthalmol. Strabismus.

[70] Beigi B., O’Keefe M., Bowell R., Naughten E., Badawi N., Lanigan B. (1993). Ophthalmic findings in classical galactosaemia—prospective study. Br. J. Ophthalmol..

[71] Luders A., Blankenstein O., Brockow I., Ensenauer R., Lindner M., Schulze A., Nennstiel U. (2021). Neonatal Screening for Congenital Metabolic and Endocrine Disorders-Results from Germany for the Years 2006–2018. Dtsch. Ärzteblatt Int..

[72] Timmers I., van der Korput L.D., Jansma B.M., Rubio-Gozalbo M.E. (2016). Grey matter density decreases as well as increases in patients with classic galactosemia: A voxel-based morphometry study. Brain Res..

[73] Timmers I., Zhang H., Bastiani M., Jansma B.M., Roebroeck A., Rubio-Gozalbo M.E. (2015). White matter microstructure pathology in classic galactosemia revealed by neurite orientation dispersion and density imaging. J. Inherit. Metab. Dis..

[74] Nelson M.D., Wolff J.A., Cross C.A., Donnell G.N., Kaufman F.R. (1992). Galactosemia: Evaluation with MR imaging. Radiology.

