# Supplementary material for: Optical Coherence Tomography: Retinal Imaging Contributes to the Understanding of Brain Pathology in Classical Galactosemia
Source: J Clin Med. 2023 Mar 3;12(5):2030. doi: 10.3390/jcm12052030 (PMC10004555; doi:10.3390/jcm12052030)
Supplement: Supplementary file 1 [file jcm-12-02030-s001.zip › jcm-2208150-supplementary.pdf]

Supplementary Figures to the article

Optical coherence tomography: retinal imaging contributes to the understanding of brain pathology in classical galactosemia

by

Amelie S. Lotz-Havla, Tara Christmann, Klaus G. Parhofer, Esther M. Maier, Joachim Havla

Supplementary Figure S1 Spectral-domain optical coherence tomography macular scan with retinal structures highlighted according to the consensus nomenclature.

Supplementary Figure S2 Baseline and follow-up spectral-domain optical coherence tomography in patient No.2.

References

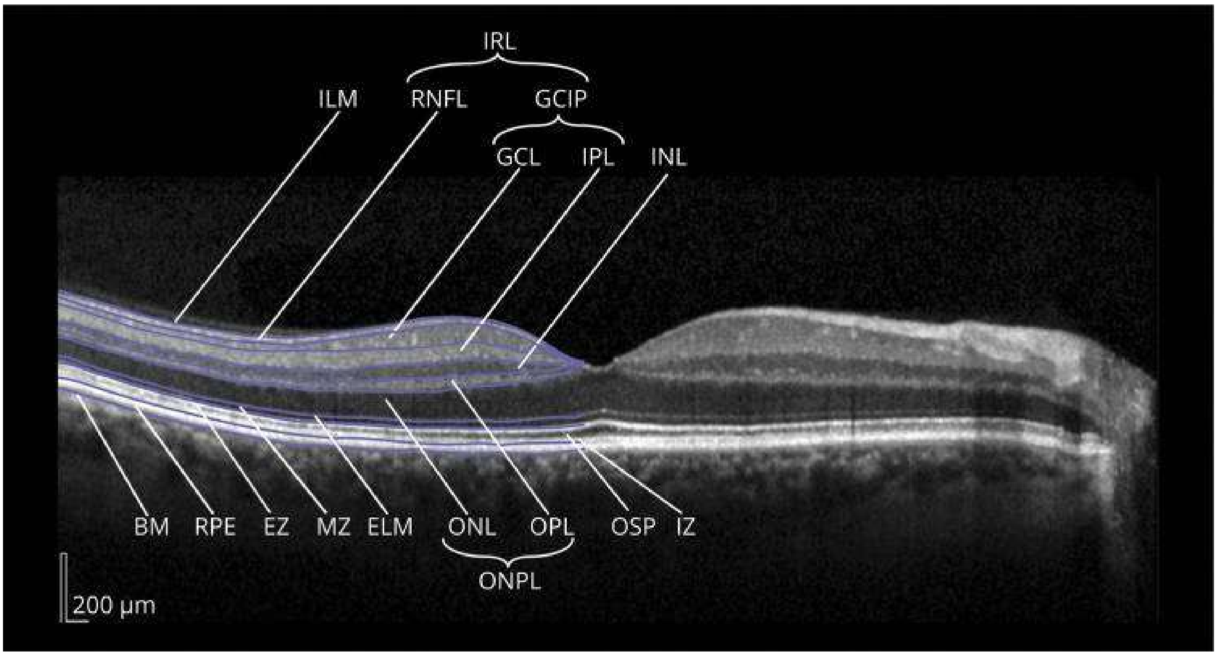

Supplementary Figure S1 Spectral-domain optical coherence tomography macular scan with retinal structures highlighted according to the consensus nomenclature [1]. Copyright is by IMSVISUAL (imsvisual.org/resources/media). Use and reprint is allowed by CC-BY-4.0. The image is unchanged. BM; Bruch membrane, ELM; external limiting membrane, EZ; ellipsoid zone, GCL; ganglion cell layer, ILM; inner limiting membrane, INL; inner nuclear layer, IPL; inner plexiform layer, IZ; interdigitation zone, MZ; myoid zone, ONL; outer nuclear layer, OPL; outer plexiform layer, OSP; outer segment of the photoreceptors, RNFL; retinal nerve fiber layer, RPE; retinal pigment epithelium. GCIP; ganglion cell and inner plexiform layer, IRL; inner retinal layers, ONPL; outer nuclear and plexiform layer.

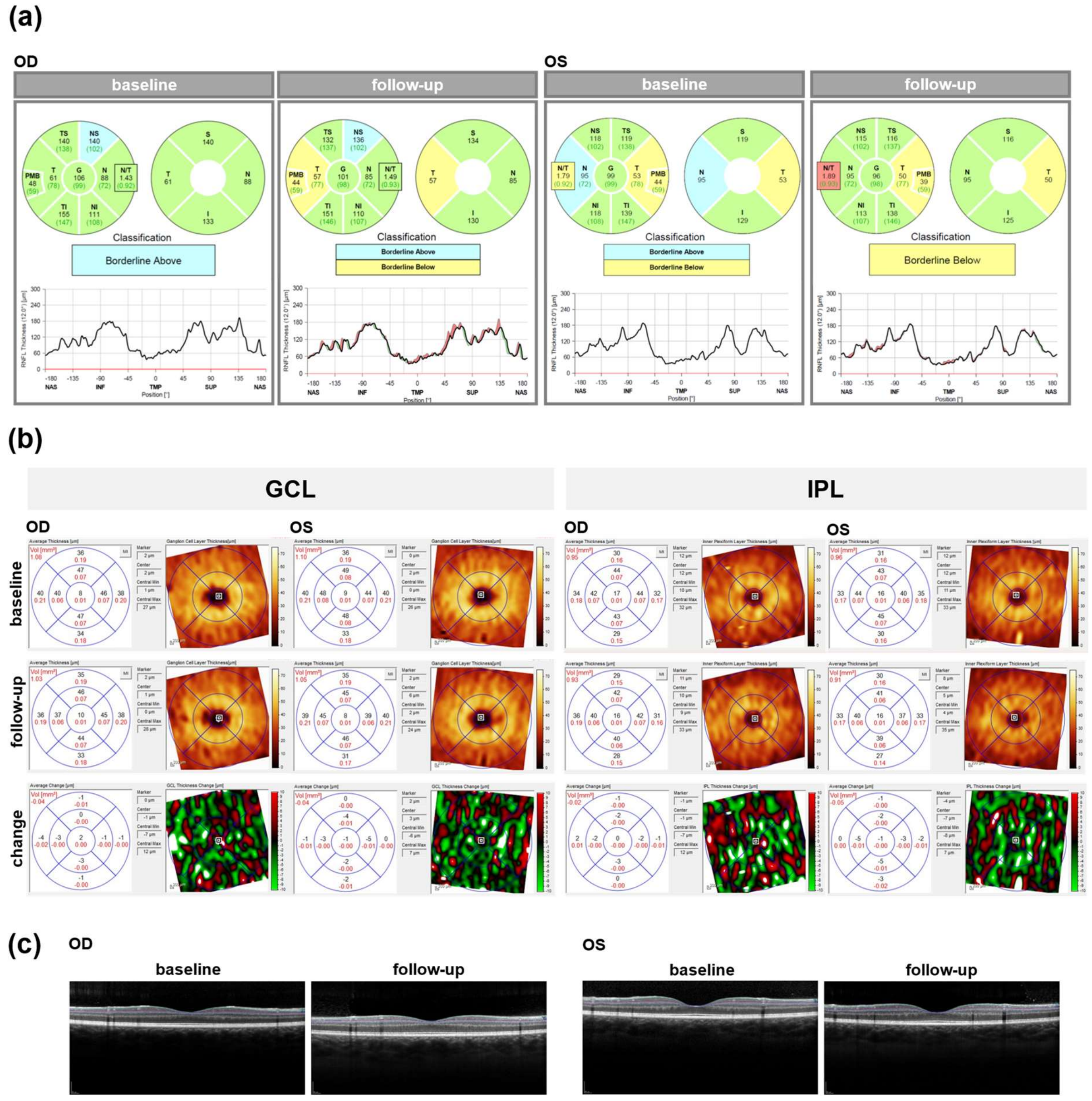

Supplementary Figure S2 Baseline and follow-up spectral-domain optical coherence tomography (SD-OCT) in patient No.2 (OD; right eye, OS; left eye). (**a**) Peripapillary retinal nerve fiber layer (pRNFL) thickness of the peripapillary quadrants (T and TMP; temporal, N and NAS; nasal, I and INF; inferior, S and SUP; superior, PMB; papillomacular bundle; G, global). (**b**) Ganglion cell layer (GCL) and inner plexiform layer (IPL) are depicted as color-coded retinal thickness map showing mean thicknesses for each of nine subfields: scan area of 6 × 6 mm, divided into three concentric circles with 1 mm, 3 mm, and 6 mm diameter. Furthermore, a retinal map is displayed showing the average change in GCL and IPL thickness from baseline to follow-up. (**c**) Baseline and follow-up macular OCT B-scans are shown, with the GCL highlighted between the turquoise and purple marker and the IPL highlighted below between the purple and blue marker.

References

1. Aytulun, A.; Cruz-Herranz, A.; Aktas, O.; Balcer, L. J.; Balk, L.; Barboni, P.; Blanco, A. A.; Calabresi, P. A.; Costello, F.; Sanchez-Dalmau, B.; DeBuc, D. C.; Feltgen, N.; Finger, R. P.; Frederiksen, J. L.; Frohman, E.; Frohman, T.; Garway-Heath, D.; Gabilondo, I.; Graves, J. S.; Green, A. J.; Hartung, H. P.; Havla, J.; Holz, F. G.; Imitola, J.; Kenney, R.; Klistorner, A.; Knier, B.; Korn, T.; Kolbe, S.; Kramer, J.; Lagreze, W. A.; Leocani, L.; Maier, O.; Martinez-Lapiscina, E. H.; Meuth, S.; Outteryck, O.; Paul, F.; Petzold, A.; Pihl-Jensen, G.; Preiningerova, J. L.; Rebolleda, G.; Ringelstein, M.; Saidha, S.; Schippling, S.; Schuman, J. S.; Sergott, R. C.; Toosy, A.; Villoslada, P.; Wolf, S.; Yeh, E. A.; Yu-Wai-Man, P.; Zimmermann, H. G.; Brandt, A. U.; Albrecht, P. The APOSTEL 2.0 Recommendations for Reporting Quantitative Optical Coherence Tomography Studies. *Neurology* **2021,** *97* (2), 68-79.
